# Elevated Hippocampal CRMP5 Mediates Chronic Stress-Induced Cognitive Deficits by Disrupting Synaptic Plasticity, Hindering AMPAR Trafficking, and Triggering Cytokine Release

**DOI:** 10.3390/ijms24054898

**Published:** 2023-03-03

**Authors:** Yu-Fen Lin, Ching-An Chen, Fang-Yu Hsu, Ya-Hsin Hsiao

**Affiliations:** 1 Department of Pharmacology, College of Medicine, National Cheng Kung University, Tainan 701, Taiwan; 2 Institute of Basic Medical Sciences, College of Medicine, National Cheng Kung University, Tainan 701, Taiwan

**Keywords:** chronic unpredictable stress, cognitive deficits, collapsin response mediator proteins, AMPA receptor trafficking, synaptic atrophy

## Abstract

Chronic stress is a critical risk factor for developing depression, which can impair cognitive function. However, the underlying mechanisms involved in chronic stress-induced cognitive deficits remain unclear. Emerging evidence suggests that collapsin response mediator proteins (CRMPs) are implicated in the pathogenesis of psychiatric-related disorders. Thus, the study aims to examine whether CRMPs modulate chronic stress-induced cognitive impairment. We used the chronic unpredictable stress (CUS) paradigm to mimic stressful life situations in C57BL/6 mice. In this study, we found that CUS-treated mice exhibited cognitive decline and increased hippocampal CRMP2 and CRMP5 expression. In contrast to CRMP2, CRMP5 levels strongly correlated with the severity of cognitive impairment. Decreasing hippocampal CRMP5 levels through shRNA injection rescued CUS-induced cognitive impairment, whereas increasing CRMP5 levels in control mice exacerbated memory decline after subthreshold stress treatment. Mechanistically, hippocampal CRMP5 suppression by regulating glucocorticoid receptor phosphorylation alleviates chronic stress-induced synaptic atrophy, disruption of AMPA receptor trafficking, and cytokine storms. Our findings show that hippocampal CRMP5 accumulation through GR activation disrupts synaptic plasticity, impedes AMPAR trafficking, and triggers cytokine release, thus playing a critical role in chronic stress-induced cognitive deficits.

## 1. Introduction

Stress arises from various sources, including work, health, family, relationships, and the economy, and these stressors can affect human health [1,2,3]. Stress includes acute stress and chronic stress. Although we often view the effects of stress negatively, the effects of stress are not always harmful. Research shows that acute stress is often beneficial for memory formation. Acute stress increases the brain’s ability to encode and recall traumatic events [4,5]. These memories are stored in the part of the brain responsible for survival and serve as warning and defense mechanisms against future trauma. However, stress can have devastating effects if it persists for a long time. Brain regions may become overstimulated during a chronic stress response, affecting cognition [6]. Acute stress can alert us and make us fight or flee from dangerous events [7,8]. Conversely, chronic stress may impair physical health and increase the risk of mental health problems, such as depression [9]. In addition, chronic stress also triggers brain inflammation, impairs memory, and impedes cognitive function, including problem solving and decision making [10,11,12]. These findings suggest that chronic stress is detrimental to cognition.

The hippocampus, a memory-related area of the brain that forms long-term memory, is involved in working memory processing and can support rapid learning [13,14]. Furthermore, the hippocampus is part of the limbic system and is particularly important in regulating stress and emotional responses [15,16]. The hippocampus is essential for spatial and episodic memory. Previous research has shown that chronic and acute stress differs in how they affect behavior and the structural integrity of the hippocampus [17]. In this study, the authors compared the effects of acute and chronic stress on neural activity in the CA1 subregion of male mice subjected to a chronic immobilization stress paradigm and observed that the spatial information encoded in the mouse hippocampus became clearer after the first exposure to stress (acute stress). However, mice exposed to chronic stress had poorer spatial attunement and decreased power of slow gamma (30–50 Hz) and fast gamma (55–90 Hz) oscillations into regions associated with excitatory input. These results show that acute and chronic stress affect hippocampal circuits differently, suggesting that acute stress may improve cognitive processing. In contrast, chronic stress causes long-term changes in the brain. Animal experiments have also found that prolonged exposure to high pressure can cause the volume of the hippocampus to shrink, which in turn affects these abilities [18,19]. However, which factor is involved in chronic stress-induced cognitive deficits is unclear.

Collapsin protein-responsive mediator proteins (CRMPs), also known as the dihydropyrimidinase-like protein (DPYSL), are a family of five intracellular phosphoproteins (CRMP1-5) with similar molecular sizes (60–66 kDa) [20,21]. Furthermore, all CRMPs consist of heterophilic oligomers that bind to tubulin and can be phosphorylated by all kinases to regulate its activity [22,23]. Studies have shown that CRMPs are highly present in the nervous system during development and in specific neuronal plasticity brain regions during adulthood, such as the hippocampus [24,25]. They play essential roles in the formation of neurite axons, the guidance of growth cones, and the interaction with microtubules.

Among the five CRMPs, CRMP1 targets RhoA signaling contributes to cytoskeletal reorganization during axonal pathfinding and is associated with neurodegeneration [26,27]. While CRMP2 regulates the stability of actin filaments, phosphorylated CRMP2 is also involved in the pathological process of Alzheimer’s disease [28]. CRMP3 is essential for dendritic elongation in neuronal differentiation and lamellipodia formation early in neurite initiation [24]. CRMP4 can bind to and regulate F-actin binding and is implicated in the neuropsychiatric field [24,29]. Furthermore, the CRMP5 protein is located on the dendrites of neuronal populations and is highly expressed during brain development. Its function is related to regulating apoptosis and differentiation, and it regulates neuronal filopodia and growth cone states [30]. Among the family members, CRMP1, 2, 3, and 4 show 75% homology, while CRMP5 has only 50% homology [31]. CRMP5 is involved in brain tumorigenesis and neurodevelopmental regulation [32,33] and is associated with several neuropsychiatric diseases [34,35]. CRMP5 expression has been reported to be increased in mouse models of stress and to accelerate memory loss in animal models of Alzheimer’s disease [24,36]. Our previous study found higher hippocampal CRMP5 expression in stress-susceptible mice than in nonstressed control and stress-resilient mice, suggesting CRMP5 can modulate susceptibility to chronic social defeat stress in mice [37]. Overall, CRMP5 is remarkable compared to other CRMP members. However, the specific role of CRMP5 in stress-induced memory impairment remains unclear.

## 2. Results

### 2.1. CRMPs Are Significantly Increased in CUS-Exposed Mice, Especially CRMP2 and CRMP5

**Figure 1:**
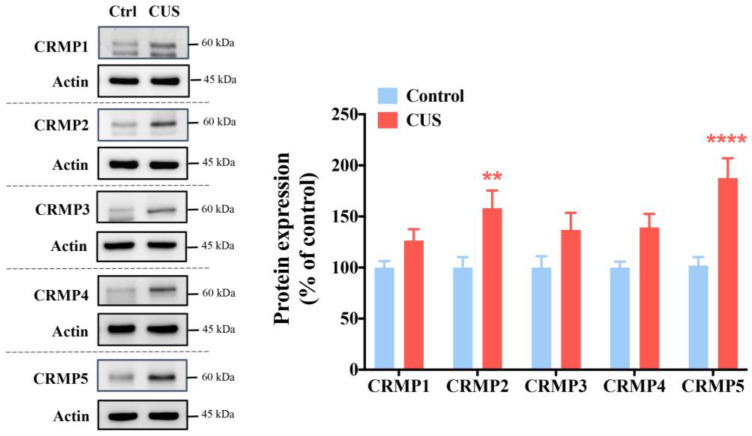
Hippocampal CRMP2 and CRMP5 levels are significantly higher in CUS-treated mice than in control mice. We collected the mouse hippocampus to assess CRMP1-5 protein levels by Western blot analysis (*n* = 16 in each group). ** *p* < 0.01 and **** *p* < 0.0001 vs. baseline of the CUS groups as determined by Bonferroni’s post hoc analysis. The data are represented as the means ± SEMs.

The CRMP family (CRMP1-5; CRMPs) is abundantly expressed in the brain, especially in the hippocampus, which modulates stress responses [24,38]. Recent studies have highlighted that CRMPs are associated with neuropsychiatric diseases, including schizophrenia [39], bipolar disorders, severe major depression, autism, and alcohol dependence [39,40,41]. Thus, we measured hippocampal CRMP protein levels in the control and CUS groups to investigate whether CRMPs are involved in CUS-induced memory impairment. The Western blot data showed hippocampal CRMP1-5 protein levels were higher than in control mice. Interestingly, among CRMPs, CRMP2 and CRMP5 levels were significantly higher in CUS mice than in nonstressed controls (Figure 1).

### 2.2. Hippocampal CRMP5 Levels Are Inversely Correlated with Memory Performance in Mice

**Figure 2:**
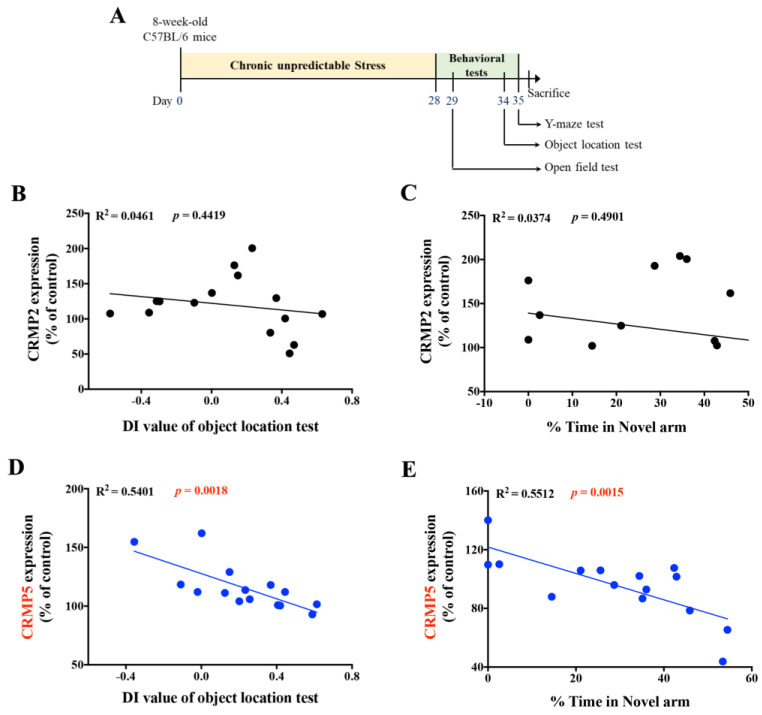
Linear regression analysis between CRMP5 expression and memory performance of mice shows a significant negative correlation. (**A**) The scheme of the experiment. Compared to CRMP2 (**B**,**C**), the percentage of hippocampal CRMP5 levels vs. the memory score plot of the object location test (**D**) or Y-maze test (**E**) showed a negative linear relationship in mice (CRMP2: R^2^ = 0.0461, *p* = 0.4419, *n* = 15; CRMP2: Y-maze test: R^2^ = 0.0374, *p* = 0.4901, *n* = 15; CRMP5: object location test: R^2^ = 0.5401, *p* = 0.0018, *n* = 15; CRMP5: Y-maze test: R^2^ = 0.5512, *p* = 0.0015, *n* = 15).

In the experiment scheme with the timeline as Figure 2A, we also calculated an association between CRMP2 or CRMP5 expression and memory performance scores of nonstressed control and CUS mice in the object location and Y-maze tests. The results showed that compared to that of CRMP2 (Figure 2B,C), CRMP5 levels had a negative linear relationship with the DI value of the object location test (Figure 2D) and the percentage of time spent in the novel arm in the Y-maze test (Figure 2E). We further confirmed that the expression of other CRMPs, CRMP1, CRMP3, or CRMP4, had no significant correlation with cognitive functions in CUS mice (Figure S1), suggesting that CRMP5 may play a critical mediator in the regulation of chronic stress-induced cognitive deficits.

### 2.3. Decreased Hippocampal CRMP5 Expression, but Not CRMP2 Expression, Ameliorates CUS-Triggered Memory Impairment

**Figure 3:**
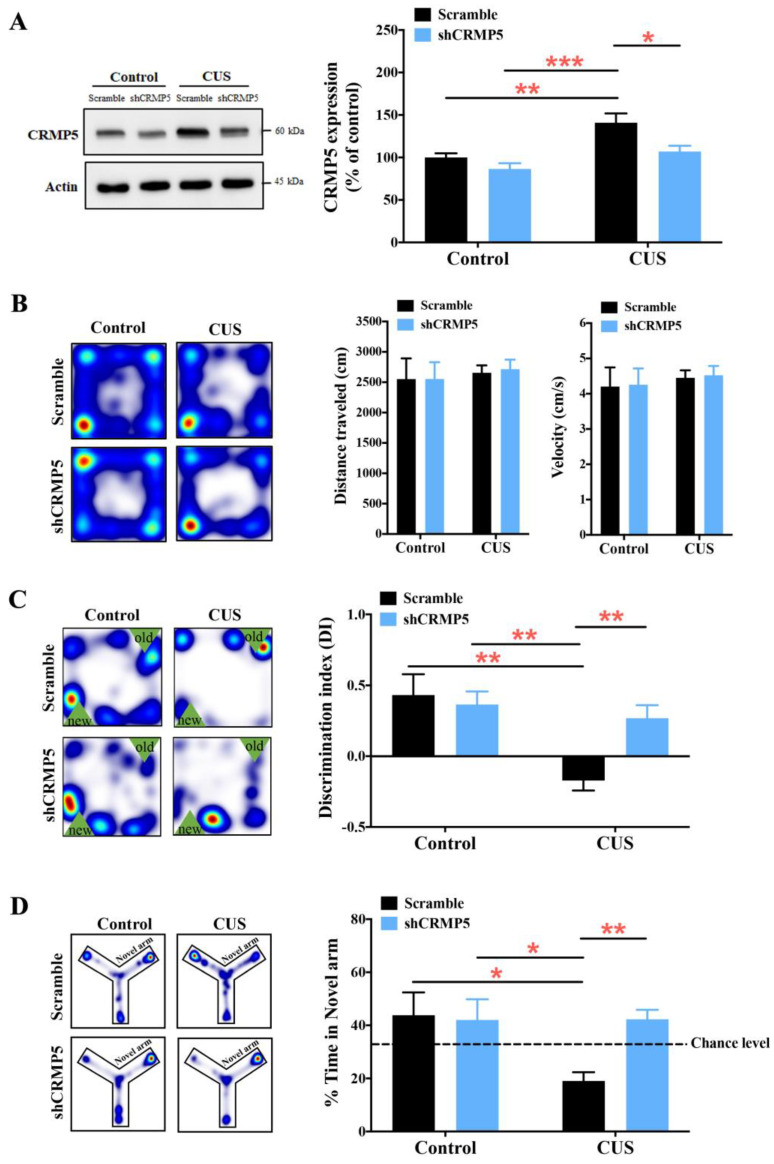
Knockdown of hippocampal CRMP5 expression reverses memory loss in CUS-treated mice. (**A**) Western blot analysis of CRMP5 expression (*n* = 7 in each group). Two-way analysis of variance revealed a significant treatment (control vs. CUS-treated mice) × shRNA interaction (*F*_(1,24)_ = 1.768, *p* = 0.1961). * *p* < 0.05, ** *p* < 0.01, and *** *p* < 0.001compared with the respective controls. (**B**) Representative heatmap of animal location during the open field test. The total distance traveled and velocity during the test were recorded. (**C**) The discrimination index (DI) indicated the time spent exploring the novel versus the familiar object location over 10 min in the object location test (control groups: *n* = 8 for the scrambled shRNA and *n* = 15 for shCRMP5; CUS groups: *n* = 10 for scrambled shRNA and *n* = 18 for shCRMP5). Two-way analysis of variance revealed a significant treatment (control vs. CUS-treated mice) × shRNA interaction (*F*_(1,47)_ = 6.176, *p* = 0.0166). ** *p* < 0.01 vs. the respective controls determined by Tukey’s post hoc analysis. (**D**) Representative heatmaps indicating time spent in the novel arm for scramble- and shCRMP5-treated mice. (Control groups: *n* = 8 for the scramble and *n* = 15 for shCRMP5; CUS groups: *n* = 10 for the scramble and *n* = 18 for shCRMP5). Two-way analysis of variance revealed a significant treatment (control vs. CUS-treated mice) × shRNA interaction (*F*_(1,47)_ = 5.379, *p* = 0.0248). * *p* < 0.05 and ** *p* < 0.01 compared with the respective controls. The data are represented as the means ± SEMs.

Next, a loss-of-function strategy was used to determine whether CRMP5 is implicated in CUS-induced memory loss. We bilaterally injected lentivirus expressing a nonspecific control shRNA (scramble) or an shRNA targeting mouse CRMP5 (shCRMP5) into the control or CUS mouse hippocampus. The data showed that the protein levels of hippocampal CRMP5 were significantly reduced in CUS mice injected with shCRMP5 compared to CUS mice injected with scrambled shRNA (Figure 3A). In addition, we also utilized the open field test to analyze mouse walking distance and velocity to check further whether the lentivirus injections would not influence mouse locomotor activity. The heatmaps and accompanying analysis data showed that scrambled- and shCRMP5-treated mice exhibited no statistical difference in distance traveled or velocity in control and CUS mice (Figure 3B). We then utilized an object location test to measure mouse memory performance. The trajectory and data of the object location test showed that shCRMP5-treated CUS mice exhibited more significant spatial memory improvement than scrambled shRNA-injected CUS mice (Figure 3C). These phenomena were confirmed by the modified Y-maze test, showing that decreased CRMP5 levels rescued memory loss in CUS mice compared to those of scrambled shRNA-treated CUS groups (Figure 3D).

Furthermore, we also knocked down hippocampal CRMP2 levels to determine whether reducing CRMP2 expression would affect memory performance in CUS mice with lentivirus expressing an shRNA targeting mouse CRMP2 (shCRMP2) or scrambled control shRNA. CUS mice transduced with shCRMP2 exhibited significantly decreased hippocampal CRMP2 protein levels, as confirmed by Western blotting (Figure S2A). In addition, there was no difference in walking distance or velocity between scrambled and shCRMP2-treated control and CUS groups (Figure S2B), showing no motor deficiency affected by virus injections. Notably, the heatmaps and accompanying analysis of object location and Y-maze test data showed that the knockdown of CRMP2 expression did not alter CUS-induced memory impairments (Figure S2C,D). The above findings highlighted that CRMP5 is indeed involved in CUS-induced cognitive deficits.

### 2.4. Enhanced CRMP5 Expression Impairs Memory Performance in Control Nonstressed Mice

**Figure 4:**
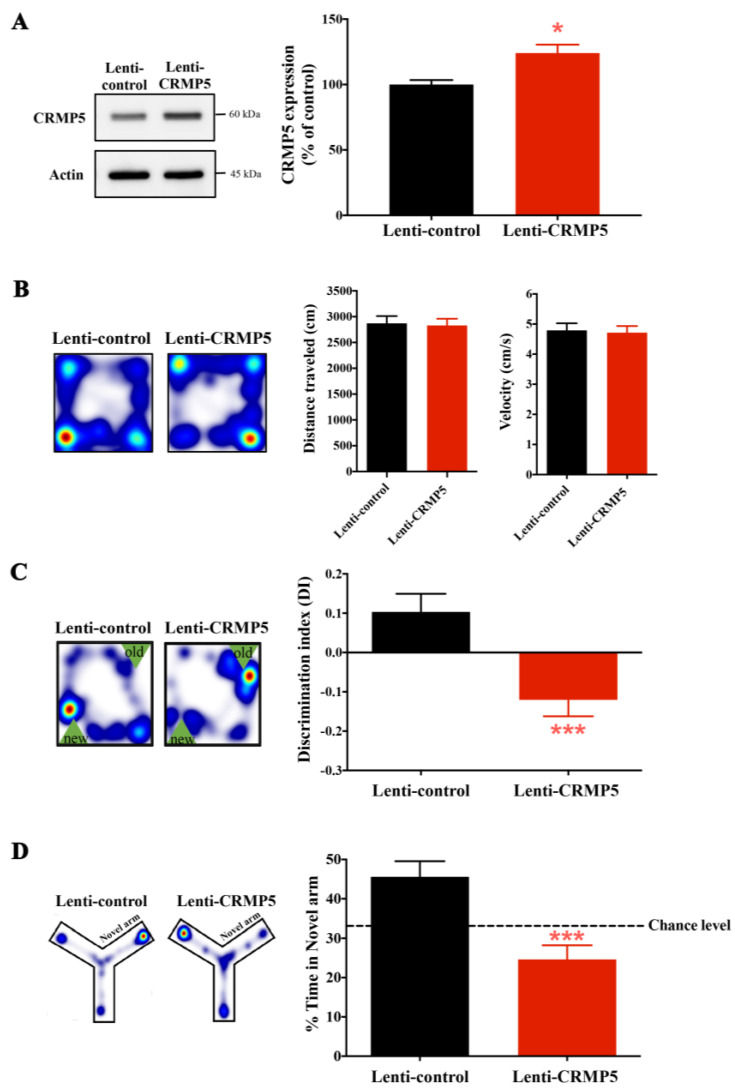
Elevation of hippocampal CRMP5 expression triggers memory decline. (**A**) Quantitative analysis of hippocampal CRMP5 expression using Western blotting. CRMP5 expression was increased in the lenti-CRMP5-treated group (*n* = 7 in each group) (two-tailed paired Student’s *t-*test; *t*_(5)_ = 3.816; *p* = 0.0124). * *p* < 0.05 vs. lenti-control group. (**B**) Representative heatmap of locomotor activity in the open field test. The distance traveled and velocity were not different between the control and lenti-CRMP5 groups. (**C**) Representative heatmaps indicate the time spent in the object location test. The DI value of the object location test showed that an increase in CRMP5 expression could cause memory impairment in lenti-CRMP5-treated mice (*n* = 20 in each group) (two-tailed unpaired Student’s *t-*test; *t*_(38)_ = 3.611; *p* = 0.0009). *** *p* < 0.001 vs. lenti-control group). (**D**) Representative heatmaps indicate the time spent in the Y-maze test. The percentage of time spent in the novel arm in the Y-maze test showed that the lenti-CRMP5-treated group spent less time than chance, indicating memory impairment in lenti-CRMP5-treated mice. (*n* = 20 in each group) (two-tailed unpaired Student’s *t-*test; *t*_(38)_ = 3.913; *p* = 0.0004). *** *p* < 0.001 vs. lenti-control group. The data are represented as the means ± SEMs.

To further confirm whether CRMP5 is involved in chronic stress-induced cognitive impairments, hippocampal CRMP5 overexpression by a lentivirus transduction strategy was applied. First, the CRMP5 cDNA clone was injected into the nonstressed mouse hippocampus to overexpress CRMP5. One month later, nonstressed mice with/without lenti-CRMP5 injection were exposed to subthreshold social defeat stress and subjected to behavioral tests. Our previous results found that the subthreshold social defeat stress could trigger social avoidance behavior but did not affect the control mice. Immunoblotting results illustrated that lenti-CRMP5-treated mice displayed higher hippocampal CRMP5 protein expression than lenti-control mice (Figure 4A). We also used the open field test and found that lenti-control and lenti-CRMP5-treated mice showed no difference in locomotor activity (Figure 4B). The trajectory analysis of the object location test and the Y-maze test showed that lenti-CRMP5-treated mice displayed memory deficits compared to lenti-control groups (Figure 4C,D), suggesting that increased hippocampal CRMP5 levels could mimic CUS-induced memory impairment in nonstressed mice.

### 2.5. Inhibiting Hippocampal CRMP5 Alleviates Chronic Stress-Induced Dendritic Atrophy and Spine Loss

**Figure 5:**
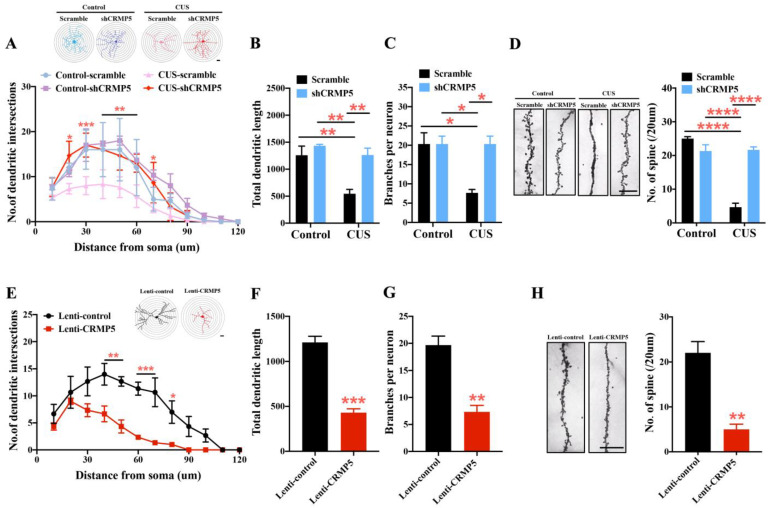
CRMP5 plays a role in modulating dendritic and spine architecture in hippocampal neurons. (**A**) Representative images (**top**) showing that inhibition of hippocampal CRMP5 by shCRMP5 rescues CUS-induced dendritic retraction. * *p* < 0.05, ** *p* < 0.01, and *** *p* < 0.001 vs. control groups. Scale bar: 50 μm. Quantitative analysis shows (**A**, **bottom**) hippocampal dendritic branching, (**B**) total dendrite length, and (**C**) branch density in the indicated groups. (**D**) Representative images (**left**) and dendritic spine number quantification (**right**). (*n* = 3 in each group). Total dendrite length: two-way analysis of variance revealed a significant treatment (control vs. CUS-treated mice) × shRNA interaction (*F*_(1,8)_ = 5.639, *p* = 0.0449). Branch density: two-way analysis of variance revealed a significant treatment (control vs. CUS-treated mice) × shRNA interaction (*F*_(1,8)_ = 9.197, *p* = 0.0162). Dendritic spine number: two-way analysis of variance revealed a significant treatment (control vs. CUS-treated mice) × shRNA interaction (*F*_(1,8)_ = 71.19, *p* < 0.0001). **** *p* < 0.0001 vs. the respective controls as determined by Tukey’s post hoc analysis. Scale bar: 5 μm. (**E**) Representative images (top) and statistical data (bottom) show that CRMP5 overexpression accelerates hippocampal dendritic atrophy. * *p* < 0.05, ** *p* < 0.01, and *** *p* < 0.001 vs. control groups. Scale bar: 50 μm. (**F**) Quantification of total dendrite length and (**G**) branch density. (**H**) Representative Golgi–Cox staining (left) and quantitative analysis of dendritic spine density (right). (*n* = 3 in each group) (two-tailed unpaired Student’s *t-*test; *t*_(4)_ = 6.140; *p* = 0.0036). ** *p* < 0.01 vs. control groups. Scale bar: 5 μm. The data are represented as the means ± SEMs.

Current theories support spinal plasticity as an integrative neurochemical structural basis for memory storage and maintenance [42,43,44]. Notably, previous studies revealed that CRMP5 is a critical factor in regulating spine plasticity [24,33]. Based on our above finding, CRMP5 expression affects CUS-induced cognitive deficits. Therefore, we evaluated whether CRMP5 alters dendritic morphology and synaptic plasticity using a Golgi staining protocol. First, we examined the impact of hippocampal CRMP5 suppression by shRNA injection on dendritic architecture. As shown in Figure 5A, dendritic branching in neurons from CUS-treated mice with shCRMP5 infusion was significantly higher than that in neurons from CUS-treated mice with scrambled shRNA injection. Branch lengths, the number of dendritic branches, and spines were also markedly higher in shCRMP5-treated CUS mice than in scrambled shRNA-treated CUS controls (Figure 5B–D), suggesting that hippocampal CRMP5 inhibition with shCRMP5 infusion alleviated CUS-induced dendritic atrophy and spine loss.

We next analyzed the effect of CRMP5 overexpression on dendrite structure in the non-CUS mouse hippocampus. Representative images and quantitative analysis of hippocampal dendritic branching are shown in Figure 5E, with more branching intersections of 40–80 μm from the neuronal soma from lenti-CRMP5-treated mice than from the lenti-control groups. In neurons overexpressing CRMP5, the total branch lengths and dendritic and spine density markedly differed from those in the lenti-control group (Figure 5F–H). These observations suggest that CRMP5 plays a role in dendritic spine remodeling.

**Figure 6:**
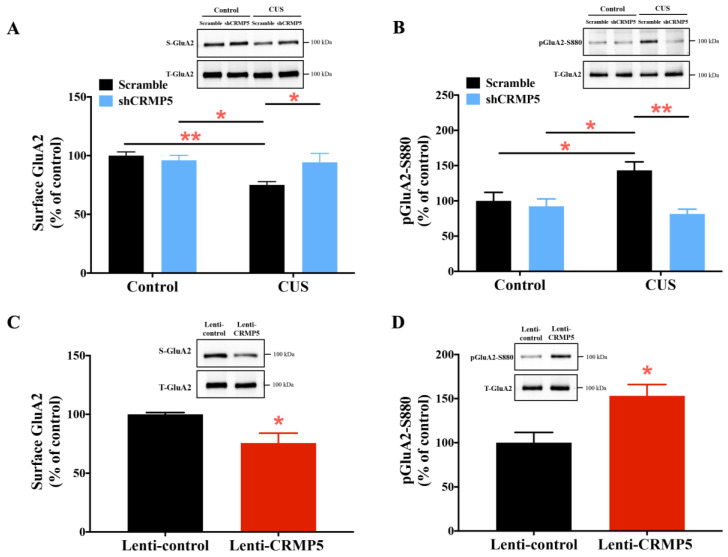
GRMP5 is a critical factor involved in hippocampal AMPA receptor trafficking. Representative Western blots and quantitative analysis show the (**A**) surface/total GluA2 ratio and (**B**) phosphorylation of S880 within GluR2 (pGluA2-S880) in hippocampal neurons from control and CUS mice without/with shCRMP5 treatment. (*n* = 6 in each group). Surface/total GluA2 ratio: two-way analysis of variance revealed a significant treatment (control vs. CUS-treated mice) × shRNA interaction (*F*_(1,20)_ = 5.729, *p* = 0.0266). pGluA2-S880: two-way analysis of variance revealed a significant treatment (control vs. CUS-treated mice) × shRNA interaction (*F*_(1,20)_ = 6.565, *p* = 0.0186). * *p* < 0.05 and ** *p* < 0.01 compared with the respective controls. Western blot analysis of the (**C**) surface/total GluA2 ratio and (**D**) pGluA2-S880 in hippocampal neurons from mice without or with lenti-CRMP5 treatment. (*n* = 6 in each group). Surface/total GluA2 ratio: (two-tailed paired Student’s *t-*test; *t*_(5)_ = 2.724; *p* = 0.0416). pGluA2-S880: (two-tailed paired Student’s *t-*test; *t*_(5)_ = 3.344; *p* = 0.0205). * *p* < 0.05 vs. control groups. The data are represented as the means ± SEMs.

α-Amino-3-hydroxy-5-methyl-4-isoxazole propionic acid receptor (AMPAR) trafficking is proposed to be linked to maintaining dendritic spine morphogenesis [45,46,47]. Furthermore, our previous study revealed that CRMP5 could regulate surface GluA2 and GluA2 S880 phosphorylation in Alzheimer’s disease-related memory impairment [36]. Thus, to further examine the underlying mechanism by which CRMP5 regulates dendritic spine remodeling, we first tested whether CRMP5 suppression rescues CUS-induced dendritic spine loss by affecting AMPAR trafficking. As illustrated in Figure 6A, there were decreased cell surface levels of the AMPAR GluA2 subunit in hippocampal neurons from CUS-treated mice. Inhibiting CRMP5 expression with shCRMP5 infusion increased surface GluA2 in hippocampal neurons from CUS-treated mice. These findings are consistent with the Western blotting results that CUS treatment promoted GluA2 S880 phosphorylation, which is involved in GluA2 endocytosis [48,49] and could be reversed with hippocampal shCRMP5 infusion (Figure 6B). Next, we analyzed how CRMP5 overexpression causes dendritic spine loss by affecting cell surface GluA2 and pGluA2-S880 expression. The Western blotting results indicated that mice with hippocampal lenti-CRMP5 infusion exhibited lower surface GluA2 and higher pGluA2-S880 protein expression than the lenti-control group (Figure 6C,D). In addition, we also verified whether manipulation of hippocampal CRMP5 levels would alter GluA1 expression. Quantitative Western blotting showed no significantly different in surface GluA1 levels among scramble- and shCRMP5-treated control and CUS mice (Figure S3A) or between lenti-control- and lenti-CRMP5-treated mice (Figure S3B). Taken together, the above immunoblotting results indicate that CRMP5 is a critical factor in mediating GluA2 trafficking.

### 2.6. Hippocampal CRMP5 Suppression Reverses Chronic Stress Triggering the Inflammatory Response

**Figure 7:**
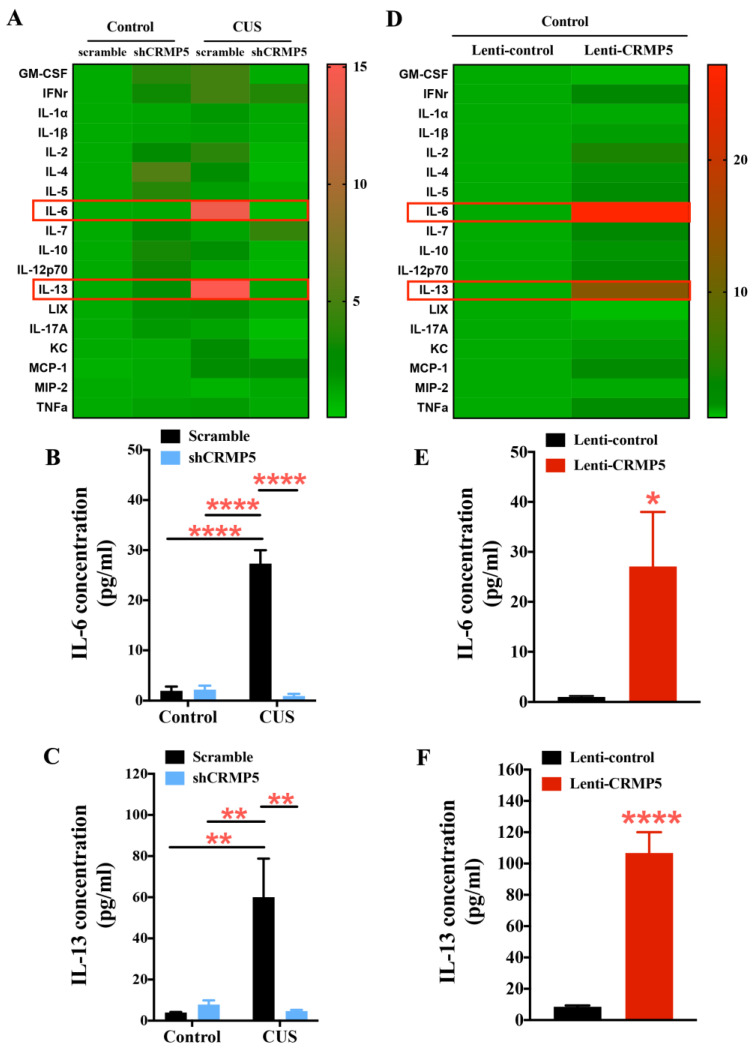
Modulating hippocampal CRMP5 expression affects the inflammatory response. (**A**) Representative heatmaps exhibit fold changes in mouse serum cytokines/chemokines from control and CUS mice without/with CRMP5 knockdown (shCRMP5). Concentrations of serum (**B**) IL-6 and (**C**) IL-13 from control and CUS mice without/with shCRMP5 treatment (control groups: *n* = 10 for the scramble and *n* = 9 for shCRMP5; CUS groups: *n* = 10 for the scramble and *n* = 10 for shCRMP5). IL-6: two-way analysis of variance revealed a significant treatment (control vs. CUS-treated mice) × shRNA interaction (*F*_(1,35)_ = 79.13, *p* < 0.0001). IL-13: two-way analysis of variance revealed a significant treatment (control vs. CUS-treated mice) × shRNA interaction (*F*_(1,35)_ = 9.392, *p* = 0.0042). ** *p* < 0.01 and **** *p* < 0.0001 compared with the respective controls. (**D**) Representative heatmaps show serum cytokine/chemokine changes in mice without or with lenti-CRMP5 treatment. Quantitative analysis of serum (**E**) IL-6 and (**F**) IL-13 from mice without or with lenti-CRMP5 treatment (n = 10 in each group). IL-6: (two-tailed unpaired Student’s *t-*test; *t*_(18)_ = 2.382; *p* = 0.0285). IL-13: (two-tailed unpaired Student’s *t-*test; *t*_(18)_ = 7.349; *p* < 0.0001). * *p* < 0.05 and **** *p* < 0.0001 compared with the control groups. The data are represented as the means ± SEMs.

To elucidate the underlying mechanisms of the involvement of CRMP5 in CUS-induced cognitive impairment, we also determined whether CUS triggers inflammatory responses that could be reduced by CRMP5 inhibition. Therefore, mouse serum from control or CUS-treated animals without/with hippocampal shCRMP5 infusion was analyzed by a mouse cytokine/chemokine panel. Consistent with our previous findings, CUS treatment may increase IL-6 and IL-13 levels [37]. Conversely, hippocampal shCRMP5 injection reversed CUS-induced proinflammatory IL-6 and IL-13 secretion (Figure 7A–C). We further studied the influence of CRMP5 overexpression on the inflammatory response, and serum from lenti-control or lenti-CRMP5 mice was analyzed. Hippocampal lenti-CRMP5 infusion triggered proinflammatory IL-6 and IL-13 cytokine release (Figure 7D–F). These findings suggest that hippocampal CRMP5 modulation systemically reduces CUS-induced proinflammatory cytokine exaggeration.

### 2.7. Chronic Stress Enhances the Phosphorylation of Glucocorticoid Receptors and Increases CRMP5 Gene Expression

**Figure 8:**
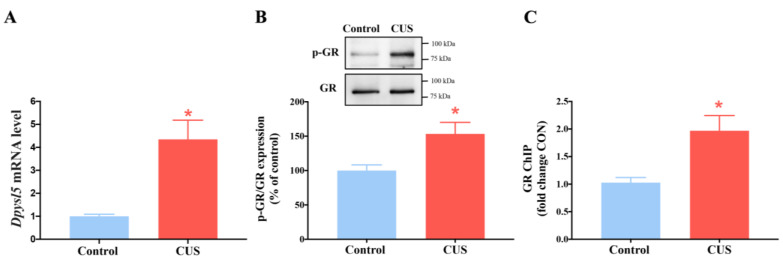
Glucocorticoid receptor is recruited to glucocorticoid receptor-binding sites to increase CRMP5 expression under chronic stress. (**A**) *Dpysl5* mRNA expression levels of the hippocampus were detected by qRTPCR (*n* = 6 in each group) (two-tailed paired Student’s *t-*test; *t*_(5)_ = 3.793; *p* = 0.0127). (**B**) Western blotting analysis shows the ratio of phosphorylation to the glucocorticoid receptor (GR) in the hippocampus from control and CUS mice (*n* = 8 in each group) (two-tailed paired Student’s *t-*test; *t*_(7)_ = 3.019; *p* = 0.0194). (**C**) Chromatin immunoprecipitation (ChIP) data revealed GR in the promoter region of Dpysl5 (CRMP5) (*n* = 8 in each group) (two-tailed paired Student’s *t-*test; *t*_(7)_ = 3.312; *p* = 0.0129). * *p* < 0.05 vs. the respective controls. The data are represented as the means ± SEMs.

We used qRT-PCR to detect mRNA expression of CRMP5 in nonstressed control and CUS-exposed mice. *Dpysl5* (CRMP5) mRNA levels in the hippocampus were higher in the CUS mice than in control mice (Figure 8A). Moreover, we identified which factor could cause the CRMP5 increase to trigger memory impairment in CUS mice. We utilized the transcription factor binding databases to screen the *Dpysl5* promoter for possible binding sites of transcriptional regulators and identified a well-conserved glucocorticoid receptor 1 (GR1, also known as NR3C1) recognition element in the proximal promoter region of *Dpysl5*. In addition, Serine (S) 211 is closely related to the activated form of GR1 (pGR1-S211) [50]. Thus, glucocorticoid and pGR1-S211 levels and the binding activity of pGR1 to the *Dpysl5* promoter were examined by Western blotting and pGR1-ChIP analysis. The immunoblotting results showed that CUS mice increased hippocampal GR phosphorylation more than the control group (Figure 8B). Next, the ChIP experiment demonstrated that the binding of GR to the promoter region of *Dpysl5* was significantly increased in the CUS-treated mice compared to the nonstressed control mice, indicating that CUS treatment increased GR transcription factor binding to the *Dpysl5* promoter, which encodes CRMP5 (Figure 8C).

## 3. Discussion

Chronic stress is a source of danger for the development of various psychiatric disorders, including depression [51,52], memory impairment [53,54,55], and even brain aging [56,57]. However, which factors cause chronic stress-induced memory deficits is still unclear. We recently demonstrated that CUS-enhanced CRMP5 expression increases in the hippocampus. To determine whether CRMP5 is involved in chronic stress-induced cognitive impairments, we manipulated CRMP5 levels by gain-of-function and loss-of-function strategies. The results showed that changes in CRMP5 expression could alter the memory performance of mice. Furthermore, modification of hippocampal CRMP5 also affects synaptic growth. Increased CRMP5 expression led to a loss of dendrites and spines. In contrast, suppression of hippocampal CRMP5 augmented the number of dendrites and spines in CUS-treated mice, which indicated that chronic stress causes hippocampal dendritic atrophy through CRMP5 regulation. The above finding aligns with previous studies showing that CUS-induced dendritic atrophy and spine loss in hippocampal neurons eventually leads to long-term potentiation (LTP) dysfunction, which is critical for learning and memory, thereby impairing memory [58,59,60].

Why was this line of mice chosen? Different strains of mice did respond differently to chronic unpredictable stress. A previous study used ICR and C57BL/6 strains of mice for comparison because they are widely used strains in behavioral tests. It was found that using the forced swimming test and novelty-suppressed feeding test, only C57BL/6 mice exhibited depression- and anxiety-like behaviors after the chronic stress procedure [61]. Thus, based on this, we also chose C57BL/6 mice for the CUS study.

Chronic unpredictable stress (CUS) is the most widely used animal model for inducing depression-like phenotypes [62]. Our previous study found that using a forced swimming test, CUS-exposed mice have depression-like behavior. We also used the chronic social defeat stress (CSDS) model, which can exhibit a pronounced depressive behavior in mice. Among the CRMP family members, we observed higher hippocampal CRMP5 expression in stress-susceptible (SS) mice than in control and stress-resilient (RES) mice [63], suggesting hippocampal CRMP5 was involved in the stress-induced depression-like behavior. In addition, chronic stress is responsible for developing many psychopathological syndromes in humans, including depression and anxiety disorders [37,64]. Previous studies revealed that rodent models of chronic unpredictable stress might induce anxiety-like behaviors [65,66]. In our tests, we did find that CUS mice also exhibited anxiety-like behaviors. Still, no correlation existed between anxiety-like behaviors and hippocampal CRMP5 expression in mice, suggesting CRMP5 expression is not associated with CUS-induced anxiety-like behaviors.

This study’s essential issue is how chronic stress could trigger CRMP5 increase to induce memory deficits. Emerging evidence has revealed that chronic stress can accelerate memory decline via the regulation of glucocorticoid receptor signaling [67,68]. Stress-induced glucocorticoid release activates the glucocorticoid receptor (GR) as a transcription factor. Phosphorylated GR (pGR) enters the nucleus and binds to the site of glucocorticoid response elements (GREs), driving gene transcription [69]. Phosphorylated GR (pGR) enters the nucleus and binds to the site of glucocorticoid response elements (GREs), driving gene transcription [69]. GR phosphorylation has been reported to negatively impact mediating morphology and function [70,71]. To confirm whether GR regulated CRMP5 expression, we measured pGR expression using Western blotting. The data revealed that pGR expression was increased after CUS treatment. We next investigated the binding activity between pGR and *Dpysl5* (CRMP5) by a chromatin immunoprecipitation (ChIP) assay and found that pGR binding to Dpysl5-GRE was significantly increased in CUS-treated mice, suggesting that chronic stress could induce an increase in pGR binding activity to trigger *Dpysl5* (CRMP5) gene expression.

Furthermore, previous studies have shown that chronic stress could promote the release of proinflammatory cytokines and chemokines [72]. To investigate whether CRMP5 affects cytokine release, CUS-treated mouse serum was collected and analyzed for inflammatory cytokines and chemokines. The serum analysis suggested that a decrease in CRMP5 levels altered the levels of proinflammatory factors. We found that CUS treatment resulted in the upregulation of proinflammatory factors, such as IL-6 and IL-13, which were then downregulated by shCRMP5 treatment. To further confirm the interaction of CRMP5 and cytokine release, we collected the Lenti-control- and lenti-CRMP5-treated mice serum to analyze many proinflammatory cytokines and chemokines. Serum analysis revealed that proinflammatory cytokines and chemokines, including IL-6 and IL-13, were upregulated in lenti-CRMP5-treated mice. Notably, hippocampal shCRMP5 injection in control mice might increase serum anti-inflammatory cytokines, such as IL-4. Modulation of CRMP5 expression can affect CUS-induced cytokine release. However, the causal relationship between CRMP5 and inflammatory factors under chronic stress remains to be elucidated.

In summary, we found that (1) chronic stress significantly increases the expression of CRMPs, especially CRMP2 and CRMP5. (2) Markedly, only the expression level of CRMP5 was closely related to the severity of memory impairment. (3) Reducing CRMP5 expression with hippocampal lentivirus delivery can rescue memory impairment in CUS-treated mice. Conversely, increasing hippocampal CRMP5 expression impairs memory in nonstressed mice. (4) Mechanistically, we also found that glucocorticoid receptor (GR), as a transcription factor, can increase the triggering of CRMP5 transcription. (5) Furthermore, regulating the expression of CRMP5 can affect synaptic atrophy and cytokine release induced by CUS. These findings suggest that chronic stress-increased CRMP5 expression is one of the leading causes of memory impairment.

## 4. Materials and Methods

### 4.1. Animals

Eight-week-old male C57BL/6 mice were obtained from BioLASCO Taiwan and housed with 3 to 5 mice per cage under standard conditions (21 ± 2 °C, 12 h:12 h light cycle) with access to food and water. Experimental procedures were approved by the Institutional Animal Care (IACUC number 109017, 110004) and Use Committee of the College of Medicine, National Cheng Kung University.

### 4.2. Chronic Unpredictable Stress (CUS)

Chronic unpredictable stress (CUS) is the most widely used animal model for inducing depression-like phenotypes [62]. The CUS procedure was performed as described [61,73] with slight modifications. CUS, a one-month stress model, consists of 10 stressors, such as food/water restriction, physical restraints, social defeat, and sleep deprivation, as our previous study described [37]. Experimental stressed mice were randomly assigned to one of the stressors every day.

### 4.3. Subthreshold Social Defeat Stress

In the subthreshold social defeat stress paradigm, male C57BL/6 mice, as intruders, were repeatedly exposed to a novel CD1 aggressor mouse for 5 min and then returned to the home cage for 15 min. The above steps were repeated three times. After subthreshold social defeat stress, the mice were triggered by social avoidance behavior but did not affect the control mice.

### 4.4. Open Field Test

The open field test was used to assess the locomotor activity of mice. We placed the experimental mouse into a 46 × 46 × 46 cm^3^ black open field box for 10 min and cleaned the box with 70% ethanol between tests. Mouse trajectory and travel distance were recorded and analyzed by EthoVision XT software (Noldus Inc., Wageningen, The Netherlands).

### 4.5. Object Location Test

The object location test is a 5-day task for assessing mouse spatial memory. First, mice were habituated into a 30 × 30 × 30 cm^3^ box for 10 min/day for 3 days. The next day, during the training phase, experimental mice were placed into the same box with two identical objects on the same side and allowed to explore for 10 min freely. On day 5, one of these two objects was placed on the opposite side of the box. Subsequently, the mice were subjected to freely exploring the objects for 10 min. Between separate trials, 70% ethanol was used to eliminate odor cues in the objects and the box in the package. The time spent exploring each object was recorded and depicted as a discrimination index (DI) value as follows: DI = (tnovel − tfamiliar)/(tnovel + tfamiliar).

### 4.6. Y-Maze Test

The Y-maze recognition test, one of the short-term spatial memory tests, was also used. First, one arm of the Y-maze was closed off, and the mice were subjected to freely explore the other two arms for 5 min. After 1 h, the closed arm opened, and the mouse was placed back into the Y maze and freely explored total arms for 5 min. The time spent in the novel arm was calculated as a percentage of the time in all three arms of the Y maze. % Time in Novel arm = [(time spent in the novel arm/total time spent in all arms) × 100].

### 4.7. Western Blot Analysis

The hippocampus of mice was dissected and sonicated in a lysis buffer. SDS—PAGE separated the extracts. The transferred membrane was blocked with 3% bull serum albumin and then incubated in primary antibodies (CRMP1 (GTX114940; GeneTex, Irvine, CA, USA), CRMP2 (ab129082; Abcam, Cambridge, UK), CRMP3 (ab36217; Abcam), CRMP4 (13661-1-AP; Proteintech, Rosemont, IL, USA), CRMP5 (GTX19352; GeneTex, Irvine, CA, USA), GluR1 (CST#13185; Cell Signaling Technology, Beverly, MA, USA), GluR2 (CST#13607; Cell Signaling Technology, Beverly, MA, USA), p-GR (CST4161; Cell Signaling Technology, Beverly, MA, USA), and NR3C1 (GR) (A2162; ABclonal, Woburn, MA, USA), and β-Actin (MAB1501; Millipore, Burlington, MA, USA) used as internal control) overnight at 4 °C. Next, after three times washing with TBST for 10 min, the membrane was incubated with secondary antibodies for 1 h at room temperature. Protein levels were detected and quantified by a BioImaging System (UVP Inc., Upland, CA, USA).

### 4.8. Golgi–Cox Staining

After the CUS challenge and behavioral test, the mice were deeply anesthetized and rapidly decapitated. Mouse brain tissues were collected, impregnated in Golgi–Cox solution for 2 days, and immersed in cryoprotectant buffer for 2 days. These tissues were sectioned into 200 μm sections, washed with 0.01 M PBST (pH = 7.4, 0.5% Triton X-100), and incubated in ammonia solution for 7 days at room temperature in the dark. The brain sections were washed with PBST, transferred to gelatin-coated slides, dehydrated with a gradient ethanol solution, and cover-slipped with a mounting medium. The synaptic and dendritic images were taken using an Olympus microscope and analyzed by ImageJ software for dendritic spine morphology.

### 4.9. Stereotaxic Intrahippocampal Lentivirus Injection

The plasmid for intrahippocampal delivery was inserted with short hairpin RNA (*Dpysl5* shRNA and *Dpysl2* shRNA or scrambled control shRNA) or lentiviruses (Lenti-control or Lenti-CRMP5 and Lenti-CRMP2) and cotransfected into HEK293 cells using Lipofectamine 2000 (Life Technologies, Carlsbad, CA). CRMP5 shRNA (*Dpysl5* shRNA) and CRMP2 shRNA (*Dpysl2* shRNA) clones were obtained from the National RNAi Core Facility (Institute of Molecular Biology, Academia Sinica, Taipei, Taiwan). *Dpysl5* and *Dpysl2* cDNA were purchased from GeneScript and Protech. Lentiviruses were collected after transfection for 48 h and then purified and concentrated to obtain 1 × 10^8^ per mL of infectious particles. The mice were mounted on a stereotactic apparatus, and two μL of lentivirus was implanted into the ventral hippocampus (anterior–posterior: −3.4 mm; medial–lateral: ± 3.4 mm; dorsal–ventral: −4.0 mm).

### 4.10. Multiplex Cytokine Assay

Blood samples were collected from sacrificed mice and centrifuged for 5 min at 2000× *g* and room temperature. The supernates from these samples were carefully collected and stored at −80 °C. For further quantifying cytokine and chemokine levels, serum samples were performed with the Multiplex Bead-Based assays following the manufacturer’s procedures (EMD Millipore; Cat: # MHSTCMAG-70KPMX, Billerica, MA, USA). Next, serum cytokine and chemokine levels were detected by Luminex^®^ (Austin, TX, USA), and the median fluorescence intensity (MFI) values of samples were assessed to calculate concentrations.

### 4.11. Chromatin Immunoprecipitation (ChIP) Assay

The mouse hippocampus was harvested and fixed with 1% formaldehyde. Chromatin immunoprecipitation (ChIP) assays were performed using a ChIP kit (Abcam, Cas: # ab500). The tissue samples were sonicated as chromatin fragments and incubated with antibodies overnight with rotation at 4 °C. Next, the chromatin/antibody samples were centrifuged (14,000× *g*, 10 min) to remove the insoluble material. Then, 250 µL of supernatant was removed, protein beads were added, and the samples were rotated at 4 °C for 1–2 h. Then, the pellet beads were precipitated by centrifugation, and the supernatant was carefully discarded. The pellet beads were treated with proteinase-K and heated to separate protein from the DNA fragments. Real-time PCR was performed using the SYBR Green-based detection system with PCR primers to measure the binding activity.

### 4.12. Statistical Analysis

All data analyses were displayed using GraphPad Prism 9. The statistical significance of differences was measured with Student’s t-test between two groups and with ANOVA for three or more groups. The values are presented as the mean ± SEM. The values are presented as the mean ± SEM, and the statistical significance was defined as *p* < 0.05.

## Data Availability

All data generated or analyzed during this study are included in this published article.

## Supplementary Materials

The supporting information can be downloaded at: https://www.mdpi.com/article/10.3390/ijms24054898/s1.
