# Supplementary material for: Elevated Hippocampal CRMP5 Mediates Chronic Stress-Induced Cognitive Deficits by Disrupting Synaptic Plasticity, Hindering AMPAR Trafficking, and Triggering Cytokine Release
Source: Int J Mol Sci. 2023 Mar 3;24(5):4898. doi: 10.3390/ijms24054898 (PMC10003309; doi:10.3390/ijms24054898)
Supplement: Supplementary file 1 [file ijms-24-04898-s001.zip › ijms-2238820-supplementary.pdf]

Supplemental Information

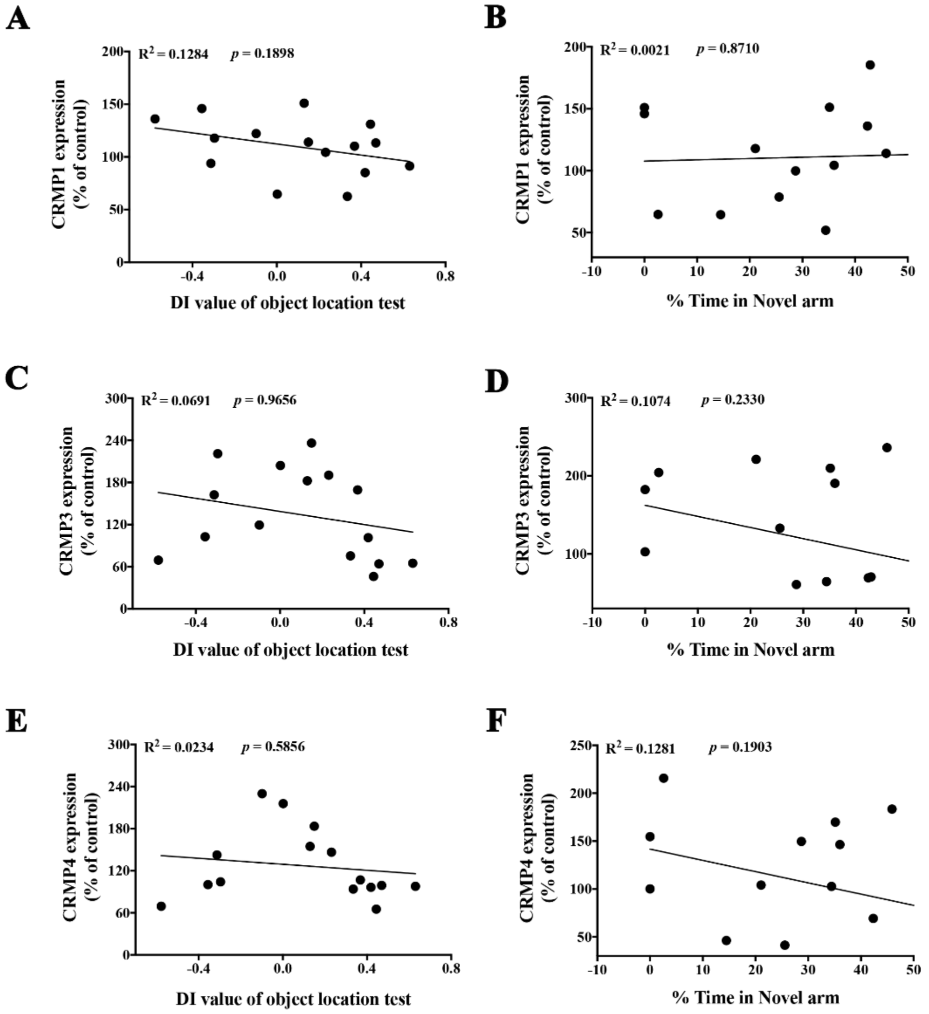

Figure S1. CRMP1, CRMP3, and CRMP4 have no significant correlation with cognitive functions in mice. (A) The correlation between CRMP1 levels and the DI value of the object location test ($R^2 = 0.1284$, $p = 0.1898$). **(B)** The correlation between CRMP1 levels and the percentage of time spent in the novel arm in the Y-maze test ($R^2 = 0.0021$, $p = 0.8710$). **(C)** The correlation between CRMP3 levels and the DI value of the object location test ($R^2 = 0.0691$, $p = 0.9656$). **(D)** The correlation between CRMP3 levels and the percentage of time spent in the novel arm in the Y-maze test ($R^2 = 0.1074$, $p = 0.2330$). **(E)** The correlation between CRMP4 levels and the DI value of the object location test ($R^2 = 0.0234$, $p = 0.5856$). **(F)** The correlation between CRMP4 levels and the percentage of time spent in the novel arm in the Y-maze test ($R^2 = 0.1281$, $p = 0.1903$).

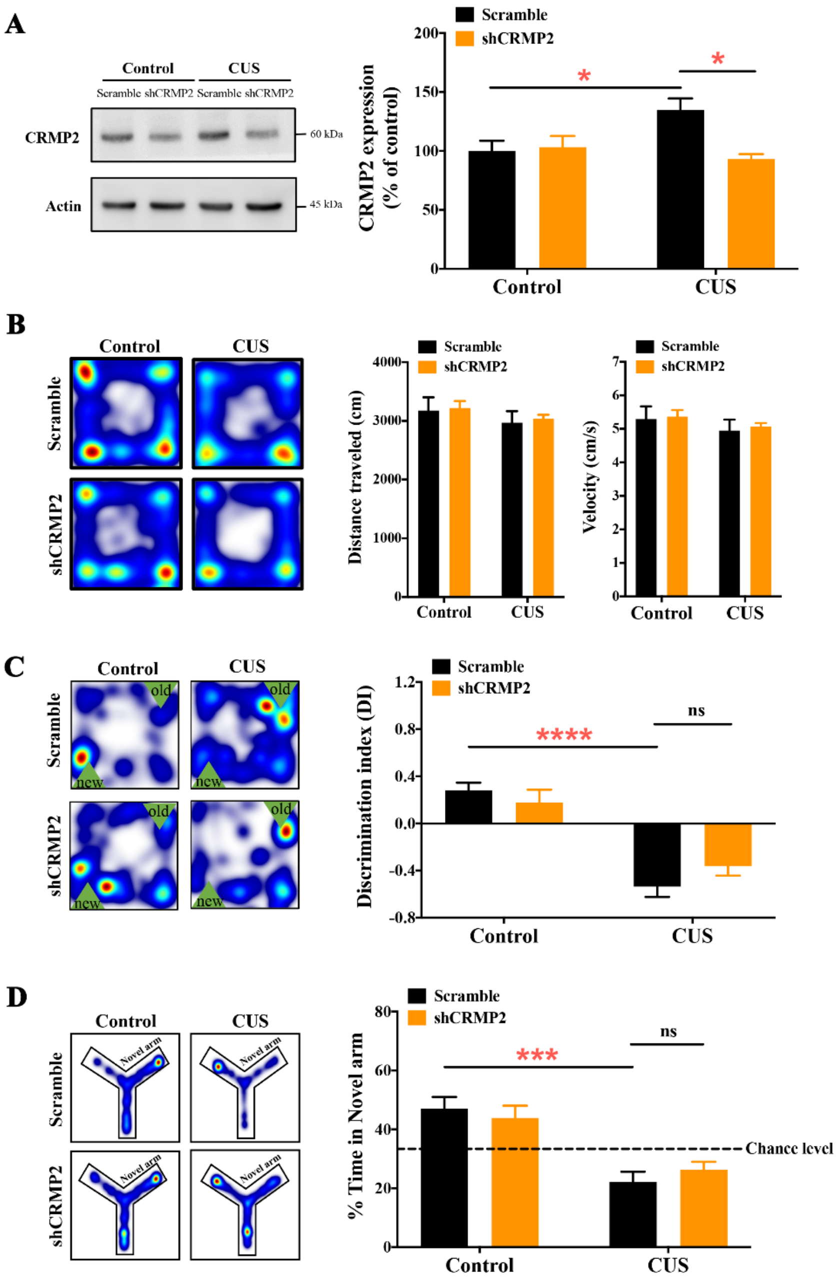

Figure S2. Reduction of hippocampal CRMP2 expression cannot alter memory impairment in CUS mice. (A) Western blot analysis of CRMP2 expression ($n = 6$ in each group). *$p < 0.05$ compared with the respective controls. **(B)** Representative heatmap of animal location during the open field test. The total distance traveled and velocity during the test were recorded. **(C)** The discrimination index (DI) indicated the time spent exploring the novel versus the familiar object location over 10 min in the object location test ($n = 8$ in each group). ****$p < 0.0001$ compared with the respective controls. **(D)** Representative heatmaps indicating time spent in the novel arm for scramble- and shCRMP2-treated mice ($n = 8$ in each group). ***$p < 0.001$ compared with the respective controls. The data are represented as the means ± SEMs.

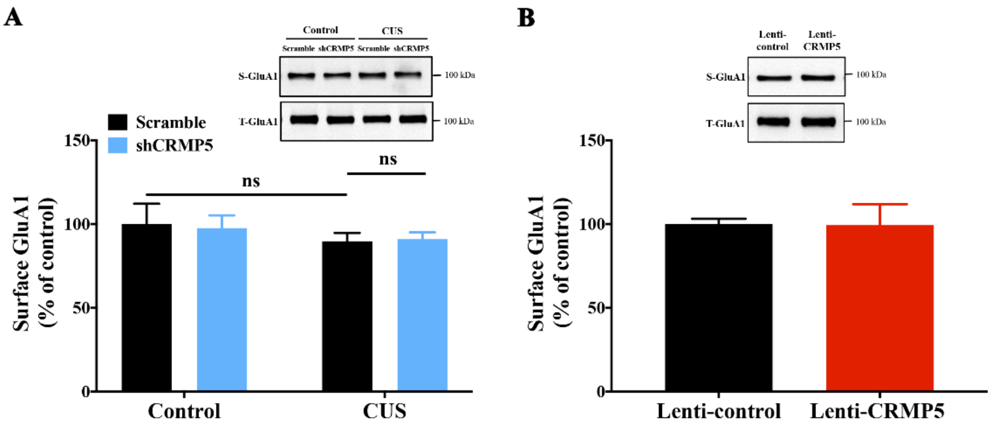

Figure S3. CRMP5 did not alter surface GluA1 levels. (A) Biotinylation assay of surface GluA1. Quantitative analysis of GluA1 expression (n = 6 in each group). **(B)** Western blot analysis of GluA2 S880 phosphorylation levels in lenti-control and lenti-CRMP5 treatments (n = 6 in each group). The data are represented as the means ± SEMs.
